# Catalytic Reduction of Environmental Pollutants with Biopolymer Hydrogel Cross-Linked Gelatin Conjugated Tin-Doped Gadolinium Oxide Nanocomposites

**DOI:** 10.3390/gels8020086

**Published:** 2022-01-28

**Authors:** Hadi M. Marwani, Shahid Ahmad, Mohammed M. Rahman

**Affiliations:** 1 Department of Chemistry, Faculty of Science, King Abdulaziz University, P.O. Box 80203, Jeddah 21589, Saudi Arabia; hmarwani@kau.edu.sa (H.M.M.); drchemsci@gmail.com (S.A.); 2 Center of Excellence for Advanced Materials Research (CEAMR), King Abdulaziz University, P.O. Box 80203, Jeddah 21589, Saudi Arabia

**Keywords:** gelatin hydrogel, Sn-Gd_2_O_3_@GH, nanocomposite, catalytic reduction, nitrophenols, azo dye

## Abstract

In the present study, a biopolymer nanocomposite hydrogel based on gelatin and tin-doped gadolinium oxide (Sn-Gd_2_O_3_@GH) was prepared for the efficient reduction of water pollutants. The method of Sn-Gd_2_O_3_@GH preparation consisted of two steps. A Sn-Gd_2_O_3_ nanomaterial was synthesized by a hydrothermal method and mixed with a hot aqueous solution (T > 60 °C) of gelatin polymer, followed by cross-linking. Due to the presence of abundant functional groups on the skeleton of gelatin, such as carboxylic acid (–COOH) and hydroxyl (–OH), it was easily cross-linked with formaldehyde. The structure, morphology, and composition of Sn-Gd_2_O_3_@GH were further characterized by the FESEM, XRD, EDX, and FTIR techniques. The FESEM images located the distribution of the Sn-Gd_2_O_3_ nanomaterial in a GH matrix of 30.06 nm. The XRD patterns confirmed the cubic crystalline structure of Gd_2_O_3_ in a nanocomposite hydrogel, while EDS elucidated the elemental composition of pure Sn-Gd_2_O_3_ powder and cross-linked the Sn-Gd_2_O_3_@GH samples. The synthesized Sn-Gd_2_O_3_@GH nanocomposite was used for the removal of different azo dyes and nitrophenols (NPs). It exhibited an efficient catalytic reduction of Congo red (CR) with a reaction rate of 9.15 × 10^−1^ min^−1^ with a strong NaBH_4_-reducing agent. Moreover, the Sn-Gd_2_O_3_@GH could be easily recovered by discharging the reduced (colourless) dye, and it could be reused for a fresh cycle.

## 1. Introduction

The presence of a low concentration of synthetic dyes is a serious threat to the reuse of industrial wastewater. Natural water sources are contaminated by heavy metals and hazardous dyes, such as nitrophenols, azo dyes, phthalates, and herbicides, with most contributions coming from oil refining and industrial discharges [1,2,3]. Dyes are mainly used in the textile, painting, leather, printing, and photography industries, among others. These dyes, after completing the dyeing process, are released as effluent to nearby water streams. The presence of these dyes creates environmental problems [4]. In particular, azo dyes contain a nitrogen–nitrogen double bond (–N=N–) and are usually applied in various industries. Various methods have been tried to remove these dyes, such as physical, chemical, and biological methods, electrochemical methods, catalysis, and adsorption [5]. Adsorption is well reported to aid in dye reduction.

Metals and their oxide nanoparticles have played the vital role of catalyst in numerous studies [6,7,8,9]. While noble metal nanoparticles have been recognized as the best catalysts for various reactions and sensing applications [10,11,12,13], their high cost limits their applications on a large scale. In contrast to noble metals, other transition metal nanoparticles, such as tin, copper, nickel, iron, and cobalt, have been reported [6,7,8,10,11,12,14,15,16,17,18,19,20,21,22] to show enhanced catalytic properties, but their stability is a major problem faced in such studies. On the other hand, transition metal oxides are quite stable in aqueous environments, and some studies have reported their use as catalysts for the degradation of dyes by electron transfer reaction [23,24,25,26,27,28]. Copper oxide, zinc oxide, ferric oxide, tin oxide, and pure metal nanoparticles could be used as catalysts, but their aggregation and separation after the reaction is complete are the main problems. Different supporting surfaces, such as block types, magnetic nanoparticles, copolymer micelles, polymer sheets, and latex particles have been used for stabilizing and easy separation [6,10,29,30,31]. Polymer hydrogels are considered to be the best for supporting catalytic nanoparticles [31]. Hydrogels are defined as cross-linked, polymeric matrix, water-swollen materials, and hydrophilic three -dimensional polymeric hydrogels have the capacity to absorb a huge amount of water and retain it without dissolution [29,31,32]. They are widely used in smart materials for controlled drug delivery, catheter coatings, tissue engineering, food chemistry, and wastewater treatment [30,33,34,35].

Rare earth elements were discovered in 1788. They consist of seventeen (17) metallic elements. In today’s society, REEs are widely used in metallurgy, high technology, military defense systems, clean energy, medical fields, fluid and auto-catalysis, wind-power turbines, electric vehicles, energy-efficient lighting, and catalytic converters [36,37]. REEs are a vital part of more than 200 products globally and many high-tech devices. Rare earth oxides (RE_2_O_3_) are most stable in their trivalent form due to their physical, electronic, and chemical properties resulting from the 4f orbital [38].

Gadolinium is a trivalent metal discovered by Johan Gadolin. Its oxide (Gd_2_O_3_) has drawn attention in the fields of garnets, magnetic resonance imaging relaxation, alloys, neutron capture, and lasers [39]. The oxide is white, odourless, and not soluble in water but soluble in acids. The oxide exists in three different forms—hexagonal (h-Gd_2_O_3_), monoclinic (m-Gd_2_O_3_), and cubic (c-Gd_2_O_3_). As a catalyst, it is used as a neutron converter in imaging plate neutron detectors, as an additive in uranium oxide (UO_2_) fuel rods and ceramics such as SiC, Si_3_N_4_, and ZrO_2_, and as a dimerization catalyst for organic compounds [40]. For the preparation of metal oxide NPs, surfactants play a vital role due to their effects on coagulation, flocculation, and particle growth [41]. Several other methods have been used for metal oxide synthesis, such as the precipitation method, the ultrasonic method, two-step solid-state reaction, spray pyrolysis, the sol-gel process, surfactant-mediated, and the hydrothermal method [42,43].

The hydrophilicity and cross-linking of polymeric chains play a vital role in investigating the properties of hydrogels and other materials. The swelling ability of hydrogels is mainly related to the hydrophilic functional group, such as carboxylic, amino acid, hydroxyl, and primary amidic, that is present in the backbone of the polymer main chain [44]. The cross-linking of this functional group between the chains makes hydrogels insoluble in water. On the basis of their swelling ability, hydrogels are categorized according to pore size. Hydrogels can be categorized as (a) non-porous, (b) micro-porous, or (c) super-porous. In non-porous hydrogels, swelling occurs through diffusion only. Micro-porous hydrogels contain pores from a few microns to several 100 micros in size, in which swelling results through diffusion as well as leaching. Super-porous hydrogels contain large-sized pores, which speed up swelling rates and, hence, cause massive swelling [45]. One serious disadvantage of such matrices is the thermal and structural lability, which can be improved with cross-linkers, such as formaldehyde [46] and glutaraldehyde [47]. In addition to their stability, hydrogels also alter swelling and, thus, trap the material inside for a longer time [48].

Therefore, biopolymers such as gelatin, chitosan, and silk proteins are widely used for hydrogel fabrications due to their essential biocompatible nature [49,50,51]. Among these polymers, gelatin has attracted the attention regarding hydrogel synthesis, due to the presence of large number of metal-binding sites on its chains [52,53]. It synthesizes industrially, playing an important role in food, photographic, and pharmaceutical industries [54]. The major sources include pig skin (46%), bovine hides (collagen) (29.4%), and cattle bones (23.1%). Gelatin composition is almost similar to collagen. Changes in its structure emerge during its manufacturing process. On heating, the collagen are transferred into helixes which, upon cooling, partly regain structure, are converted into gelatin containing water, and form gel. However, due to its brittle and fragile network, it is limited in application. To control these limitations, MNPs have been introduced to form hydrogel nanocomposites [55]. The entrapped NPs either cross-link the chains of the hydrogel or absorb on the polymer chain, forming a three-dimensional network [56]. These networks are resulted due to covalent and noncovalent bonds through physical or chemical interactions. The MNPs and polymer work together to produce unique properties, such as thermal, mechanical, and optical, which leads the hydrogel nanocomposites to be used in sensors, catalysts, electronics, optics, and in separation devices [57]. Gelatin hydrogel can also be used as biodegradable material in the pharmaceutical and medical industries [56,58,59]. Moreover, it can be cross-linked easily due to the presence of a large number of functional groups [60].

Therefore, herein, the gelatin hydrogel nanocomposite was prepared by the incorporation of gadolinium doped tin dioxide nanomaterials and cross-linked with formaldehyde. The prepared hydrogel nanocomposite was studied as a catalyst for the reduction of different environmental pollutants. Additionally, the prepared hydrogel exhibits low cost, highly efficiency for the removal of dyes, potential recyclability, and easy separation.

## 2. Experimental

### 2.1. Materials

**Scheme 1:**
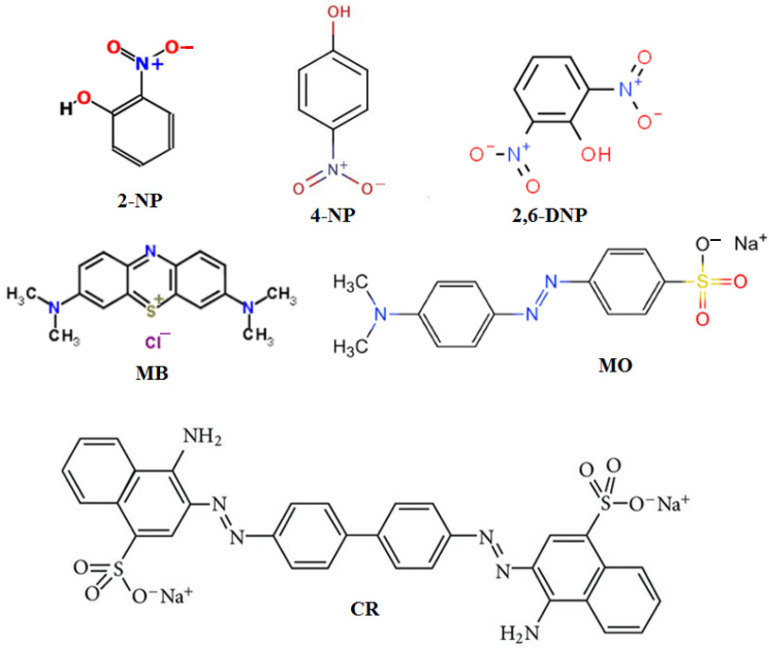
Molecular structures of 2–NP, 4–NP, 2,6–DNP, MB, MO, and CR.

The chemicals and reagents used this experiment were analytical grade. Gelatin (C_102_H_151_O_39_N_31_), 4-nirophenol (4-NP–C_6_H_5_NO_3_), 2-nitrophenol (2-NP–C_6_H_5_NO_3_), 2,6-dinitrophenol (2,6-DNP–C_6_H_4_N_2_O_5_), and methylene blue (MB; C_16_H_18_ClN_3_S) were purchased from Fluka, UK. Aridine orange (ArO; C_17_H_19_N_3_), congo red (CR-C_32_H_22_N_6_Na_2_O_6_S_2_), and methyl orange (MO; C_14_H_14_N_3_NaO_3_S) were purchased from Sigma-Aldrich chemical company (Austin, TX, USA). Reducing agent, sodium borohydride (NaBH_4_, 97%), was purchased from BDH chemicals, Poole, United Kingdom. Ultra-pure distilled water was used for the preparation of all the solutions. The molecular structure of all target chemicals and dyes such as 2-NP, 4-NP, 2,6-DNP, MB, MO, and CR are given in Scheme 1.

### 2.2. Synthesis of Sn-Gd_2_O_3_ Nanomaterial

Gadolinium is a vital substance for doping due to its chemical stability. Here, a one-step way of fabrication is followed for Sn-Gd_2_O_3_ nanomaterial. In the first step, 0.1 M solution of gadolinium salt [Gd(NO_3_)_3_·6H_2_O] was prepared, dissolving 2.26 gm in 50 mL of distilled water. Similarly, 0.01 M solution of SnCl_4_ salt (0.1303 gm) was freshly prepared in 50 mL of ethanol. The SnCl_4_ solution was set on vigorous stirring for 1hr, until a colourless solution was obtained. In the second step, both solution [Gd(NO_3_)_3_·6H_2_O] and SnCl_4_ was mixed up in 100 mL of round bottom flask. The pH of the final solution was adjusted above 10 by adding drop wise NH_4_OH solution. Again, the solution was kept for stirring, forming colloidal suspension, after 1 h. Finally, the solution was transferred to a Teflon-lined autoclave at 150 °C for 3 h, which gives white precipitate. The final product was collected after centrifugation. The resulting white product was washed with water and ethanol and dried at 80 °C overnight.

### 2.3. Preparation of Gelatin Hydrogel Solution

Gelatin hydrogel was prepared by dissolving of 2.4 gm of gelatin in 30 mL of distilled water, at room temperature. The beaker containing hydrogel was heated mildly at 60 °C, under constant stirring, to prepare a homogenous viscous solution of gelatin polymer.

### 2.4. Fabrication of Sn-Gd_2_O_3_@GH Nanocomposite

**Figure 1:**
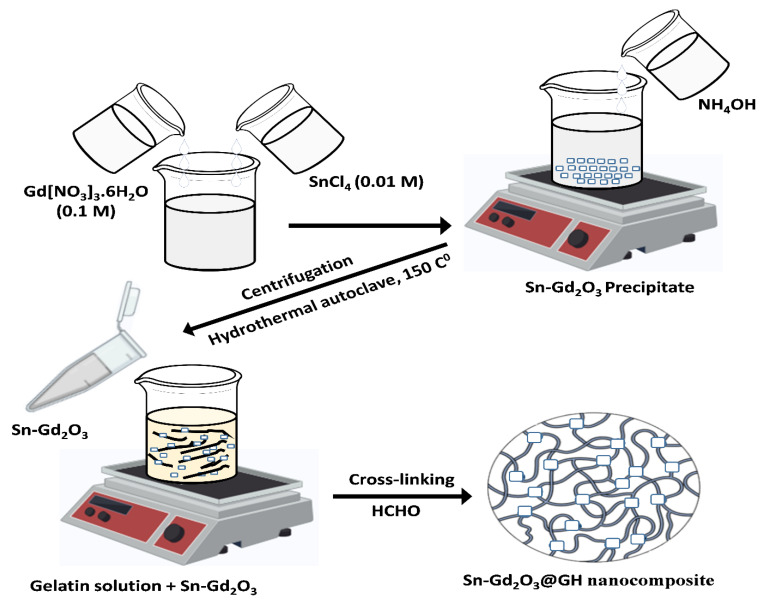
Preparation of Sn-Gd_2_O_3_@GH nanocomposite.

During adjustments, the 8 wt% concentration of gelatin was found to be good. Thus, 20 mL of gelatin solution was used to prepare hydrogel nanocomposite. The hydrogel was further treated with 4% by weight of Sn doped gadolinium oxide (Sn-Gd_2_O_3_) in a beaker under constant stirring for 40 min, to completely homogenize the Sn-Gd_2_O_3_ nanomaterial. The hydrogel nanocomposite was cross-linked by drained off slowly 5 mL of formaldehyde and left for 24 h to completely cross-link the hydrogel. The prepared solid hydrogel was mashed out and washed three times with distilled water to remove the surplus Sn-Gd_2_O_3_ NPs (Figure 1).

### 2.5. Preparation of Dyes Solution

All of the dyes, 4-NP, 2-NP, 2,6-DNP, ArO, MO, and CR, were used. Standard solutions were prepared with desired concentrations of 0.07 mM. Ultra-pure distilled water was used throughout all the experiments.

## 3. Characterizations

In this approach, scanning electron microscopy, (FESEM) of JEOL, JSM-7600F, Tokyo, Japan, was used for the morphological investigation of Sn-Gd_2_O_3_@GH nanocomposite, at high accelerating voltage. The prepared sample was coated with platinum before analysis, and attached on 1.5 cm diameter of sticky black tape. The surface elemental composition of the sample was analysed using Oxford energy dispersive X-ray spectroscopy (EDS; Tokyo, Japan) equipment, attached with FESEM (Tokyo, Japan). X-ray diffraction (XRD) experiments were conducted on PANalytical diffractometer using Kα radiation (λ = 0.154 nm) source to investigate the crystallinity of the samples. The voltage of 40 KV and current of 50 mA was set for analysis. The analysis data was recorded in the range of 10° and 80° at a scan rate of 2° 2θ min^−1^. Fourier transform infrared (FTIR; Berlin, Germany) was used to characterize different functional groups in the Sn-Gd_2_O_3_@GH nanocomposite hydrogel. The spectra were recorded in the range of 40,000 to 500 cm^−1^ by averaging the 64 scans, using FTIR (Berlin, Germany).

### 3.1. FESEM Analysis

Field Emission Scanning Electron Microscope (FESEM; Tokyo, Japan) is an electron microscope which is suitable for the analysis of material surface. It is used for observing structures as small as 1 nm. For sample analysis, the primary electrons from a field source are focused and deflected by the lenses which produce a narrow scan beam that investigates the surface morphology of the sample. The secondary electrons are reflected from the material surface to the detectors, which transform it to an electronic signal.

**Figure 2:**
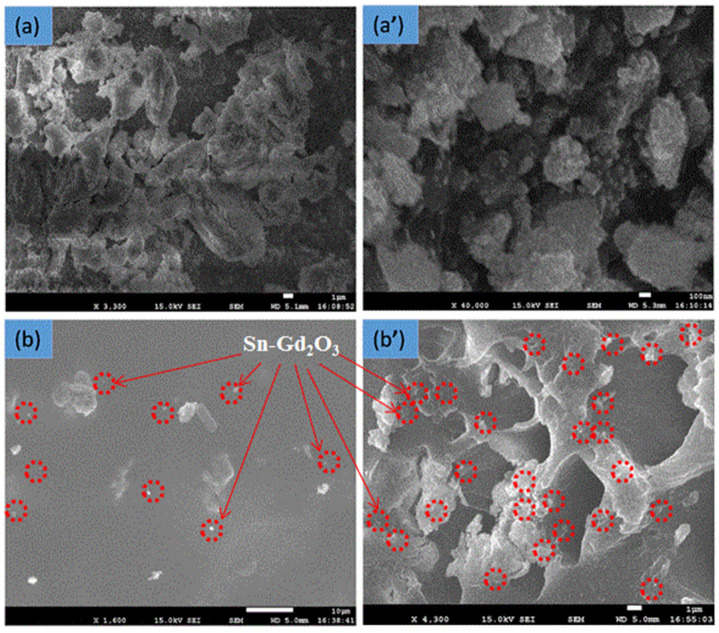
FESEM images of pure Sn-Gd_2_O_3_ (**a**) and Sn-Gd_2_O_3_@GH (**b**) at low and their high magnification images (**a’**), (**b’**).

FESEM is used to analyse the surface morphology of the samples, usually for the exterior and cross-sectional area. Figure 2a,b shows the FESEM images of powder Sn-Gd_2_O_3_ and Sn-Gd_2_O_3_@GH nano-composite, respectively. The Sn-Gd_2_O_3_ particles are clearly seen in irregular form as shown in Figure 2a, while, Figure 2b confirms the dispersed nature of Sn-Gd_2_O_3_ nanomaterials (red circle on SEM image) in the hydrogel matrix. Figure 2b,b′ shows the as synthesized Sn-Gd_2_O_3_@GH nano-composite present in the white aggregated form. The average particle size of 30.06 nm was calculated. The incorporation of Sn-Gd_2_O_3_ nanomaterial (red circle on SEM image) changed the morphology of the gelatin polymer; it appeared in grooves and wide roughness, as shown in Figure 2b**′**.

### 3.2. EDX Analysis

**Figure 3:**
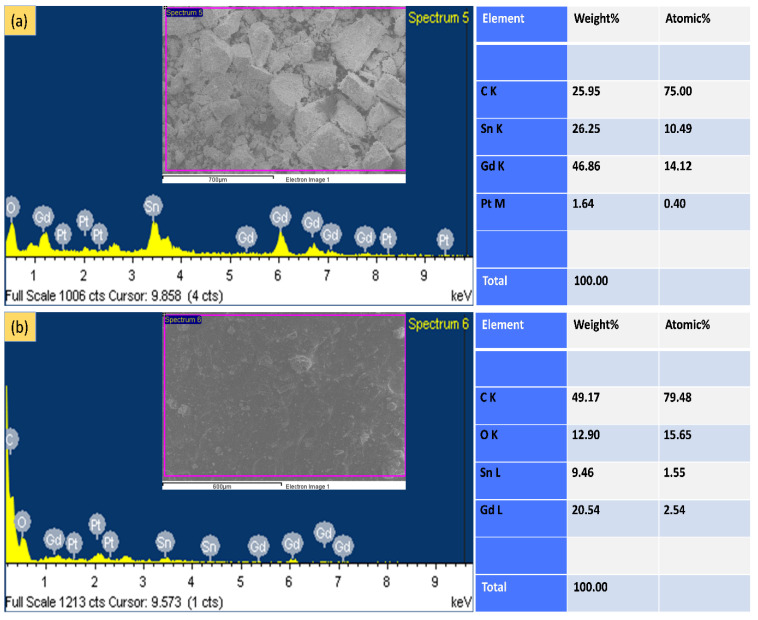
EDX spectra of Sn-Gd_2_O_3_ nanomaterial (**a**) and Sn-Gd_2_O_3_@GH nanocomposite (**b**).

Energy dispersive-ray spectroscopy is a surface microanalysis characterization to analyse the elemental composition of the sample. Figure 3 elucidates the EDX spectra of Sn-Gd_2_O_3_ and Sn-Gd_2_O_3_@GH nanocomposite. EDX spectra showed the existence of Sn, Gd, and O elements into the Sn-Gd_2_O_3_ doped nano-catalyst (Figure 3a). In another EDX spectrum, it is found the Sn, Gd, O, and C elements into the gelatin polymer coated Sn-Gd_2_O_3_@GH nanocomposite (Figure 3b). The platinum peak showed due to the platinum coating on the sample. The former EDX spectra for Sn and Gd revealed 26.25 % and 46.86 % by weight, while the second spectra showed 9.46 % and 20.54 % by weight amount of Sn and Gd, respectively. Figure 3b showed the elemental composition of Sn-Gd_2_O_3_ nanomaterial in the hydrogel matrix. This homogenous distribution confirms the Sn-Gd_2_O_3_ in the hydrogel nanocomposite.

### 3.3. XRD Analysis

**Figure 4:**
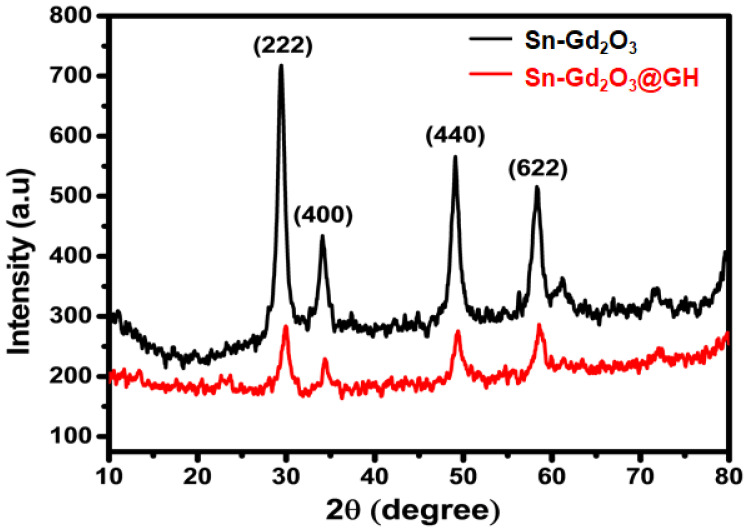
XRD patterns of Sn-Gd_2_O_3_ nanomaterial (Black-line) and Sn-Gd_2_O_3_@GH nanocomposite (Red-line).

The XRD profiles of powder Sn-Gd_2_O_3_ nanomaterial and Sn-Gd_2_O_3_@GH nanocomposite are presented in Figure 4. Figure 4 (Black-line) shows the XRD profile of the Sn-Gd_2_O_3_ nanomaterial having four sharp peaks. The pattern of Gd_2_O_3_ powder is exactly coincident with cubic phase Gd_2_O_3_ (JCPDS card no.12-0797), as published earlier in the related literature [61]. The prominent peaks appeared at 2-theta of 29.39°, 34.12°, 49.12°, and 58.33°, and could be assigned to (222), (400), (440), and (622) diffraction of Sn-Gd_2_O_3_ nanomaterials, respectively. Figure 4 (Red-line) showed corresponding peaks for Sn-Gd_2_O_3_@GH nanocomposite and calculated no substantial difference in the XRD pattern of the Sn-Gd_2_O_3_ nanomaterial sample, except a decrease in the intensity of the peaks. No significant peaks were observed for the dopant due to its low concentration.

### 3.4. FTIR Analysis

**Figure 5:**
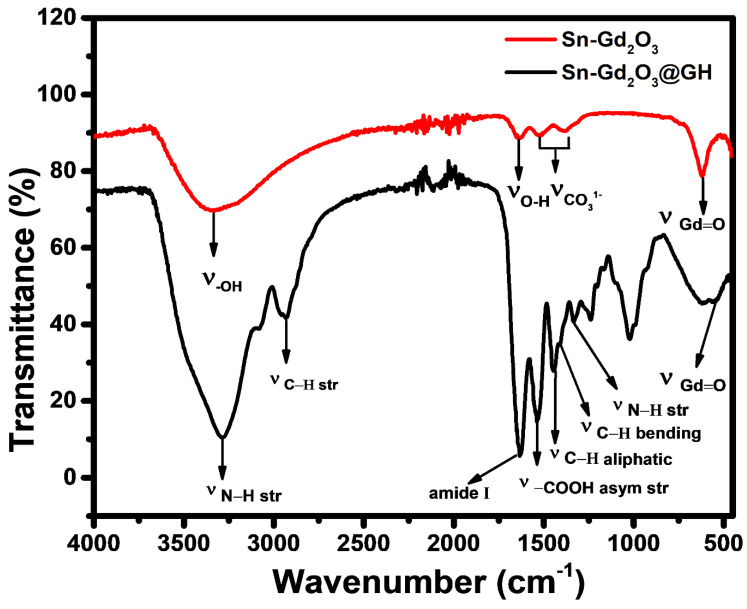
FTIR spectra of Sn-Gd_2_O_3_ nanomaterial and Sn-Gd_2_O_3_@GH nanocomposite.

FTIR is used to investigate the chemical composition of the sample surface, examine the different functional groups residing in the molecular structure of the compound, and identify peaks change in the mixed substances. Herein, the spectra were systematically recorded in the range of 500–4000 cm^−1^ for the sample analysis. Figure 5 shows the ATR–FTIR spectra of Sn-Gd_2_O_3_, and dried Sn-Gd_2_O_3_@GH nanocomposite film. In the FTIR spectrum of powder Sn-Gd_2_O_3_, the characteristic peaks at 1643, 1513, and 1379 are attributed to the –OH vibration of water and asymmetric stretching vibrations of the carbonate group which may be due to the presence of the air [62], the two prominent peaks exhibited at 3322 and 618 cm^−1^ that are related to the symmetric stretching vibration of –OH, and the Gd=O vibrational band of Sn-Gd_2_O_3_. Similarly, many peaks appeared in the FTIR spectra of Sn-Gd_2_O_3_@GH nanocomposite. The peaks observed at 3284 and 2930 cm^−1^ were attributed to the stretching of N–H functional and C–H stretching vibration of gelatin structure. The peak at 1636 cm^−1^ is present amid I; the peak at 1409 cm^−1^ is attributed to the C–H bending vibration; while peaks appearing at 1438 and 1537 cm^−1^ suggest C–H aliphatic vibration and asymmetric stretching vibration of carboxyl group, respectively. Similarly, the peak which appeared at 1337 is attributed to the N–H stretching vibration. Moreover, the broader peak was appeared for Gd=O, at 553 cm^−1^, which is similar to published report [63], and confirm the presence of a Sn-Gd_2_O_3_nanomaterial in the hydrogel nanocomposite. In FTIR spectrum of powder Sn-Gd_2_O_3_, the characteristics peaks at 1643, 1513, and 1379 are attributed the stretching vibration of hydroxyl and asymmetric stretching vibrations of carbonate group, which may be due to the presence of the air.

## 4. Result and Discussion

### 4.1. Dyes Reduction

The catalytic activity of Sn-Gd_2_O_3_ NPs was analysed against two series of different dyes, such as azo dyes, and different NPs. For each dye, 25 mL of (0.07 mM) of each MB, MO, and CR were added into a 50 mL beaker with strong reducing agent NaBH_4_ under constant stirring. The reduction of the dyes was investigated by introducing 0.4 g of NaBH_4_, which suddenly brought a small change in the colour of the dyes. A catalyst of 0.2 gm (Sn-Gd_2_O_3_@GH) was added to the beaker promptly after adding the NaBH_4_. The catalytic reduction was recorded through UV–visible spectrometer. For the recording of every spectral line the dye was introduced to quartz cuvette cell of 3.5 mL. For the reduction investigation, tiny amount of the aliquot was taken out in the beaker in quartz cuvette for spectral analysis. The cuvette solution is drained off into the beaker again and continued the process until completion of the reaction. The hydrogel nanocomposite reduced the MO and CR in 16 and 4 min, respectively. No change in the spectra of MB was observed for 20 min. Similarly, different nitro phenols, such as 4-NP, 2,6-DNP, and 2-NP, were investigated following the same process as discussed above. Hence, the 4-NP, 2,6-DNP, and 2-NP nitrophenols were reduced in 16, 12, and 8 min, respectively.

Therefore, the catalytic efficiency of Sn-Gd_2_O_3_@GH catalyst reduced the MO, CR, 4-NP, 2,6-DNP, and 2-NP, among which the CR was reduced efficiently in 4 min. The concentration of all of the dyes was 0.07 mM, using 0.4 gm of NaBH_4,_ and 0.2 gm of the catalyst throughout the experiment.

#### 4.1.1. Catalytic Reduction of NPs

The catalytic activity of Sn-Gd_2_O_3_@GH nanocomposite was investigated against 4-NP, 2,6-DNP, and 2-NP with of strong reducing agent (NaBH_4_), as model reactions. UV–visible absorption spectra were recorded verses irradiation time to investigate the colour changes in the reaction phase. It was observed that the addition of 0.4 g of strong reducing agent to different nitrophenol solutions caused an immediate change in their colour. However, no decrease in the intensity at main peaks was observed. Upon 0.2g of Sn-Gd_2_O_3_@GH nanocomposite addition as a catalyst to the solutions, the NPs were reduced. For each reaction, 25 mL of dye solution (0.07 mM) was added to a 50 mL beaker, proceeding with the addition of 0.4 gm NaBH_4_ and 0.2 gm catalyst under constant stirring.

**Figure 6:**
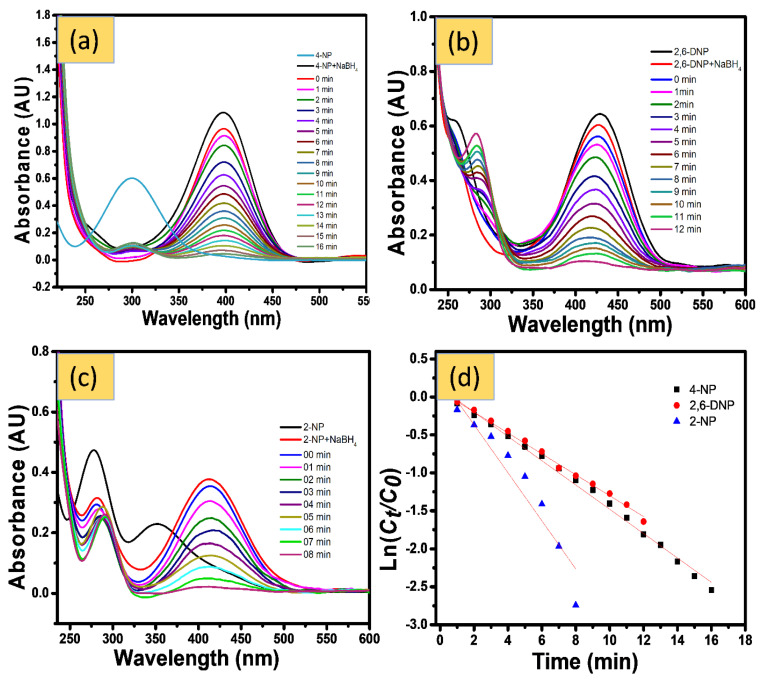
Typical UV−visible absorbance spectra of 4−NP (**a**), 2,6−DNP (**b**), and 2−NP (**c**) and their (**d**) Ln(At/A0) vs. time plot for the reduction reactions where the amount of the Sn-Gd_2_O_3_@GH catalyst was 0.2 g.

Figure 6a shows the catalytic reduction of 4-NP to (4-aminophenol) 4-AP with reducing agent NaBH_4_. The aqueous solution of 4-NP showed a maximum absorption peak (λ _max_) at 317 nm, which red shifted to 398 nm, with the addition of NaBH_4_. This shift confirms the formation of 4-nitropnolate ions as described in some previous reports [64,65]. Due to the addition of 0.2g hydrogel catalyst, the intensity of the peak at 398 nm starts decreasing with the concomitant increase in 4-AP peak at 300 nm. Therefore, the light yellow solution of 4-NP had disappeared after 16 min. A similar procedure was followed for the 2,6-DNP and 2-NP (0.07 mM), as shown in (Figure 6b,c).

Their peaks appeared at 428 nm and 413 nm were reduced in 12 and 8 min, respectively. In these catalytic reactions, the concentration of NaBH_4_ was much higher and can be considered constant. Kinetic study was proposed to investigate the mechanism of adsorption process of the dye on the contact surface of the catalyst. To quantitatively measure the rate of these redox reactions, the UV–vis spectral data was calculated through the following pseudo-first-order kinetics.

Ln (*C_t_/C*_0_) = Ln(*A_t_/A*_0_) = −*K*_app_ t (1)

where *C*_0_ is the initial concentration of the dye and *C_t_* is the concentration of the dye at any given interval of time. *A*_0_ and *A_t_* are the absorbance value take before and after introducing NaBH_4_, respectively, and catalysts to the reaction media. *K*_app_ represent the apparent rate constant, while t represents the rate of the reaction.

Therefore, the catalytic efficiency of Sn-Gd_2_O_3_@GH catalyst on the reduction of 4-NP, 2,6-DNP, and 2-NP can be described as pseudo first-order. The reduction rates of 4-NP, 2,6-DNP and 2-NP were reported as 1.6 × 10^−^^1^, 1.4 × 10^−^^1^, and 1.8 × 10^−1^ min^−^^1^, respectively.

#### 4.1.2. Catalytic Reduction of Azo Dyes

**Figure 7:**
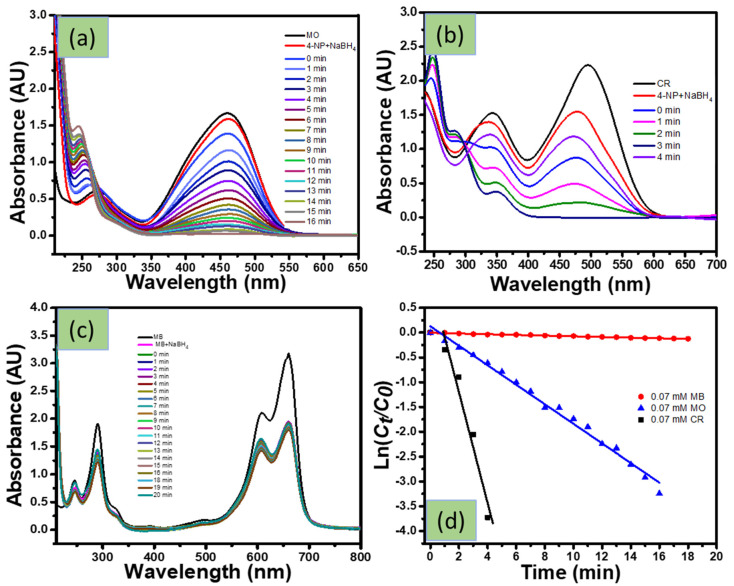
Typical UV–visible absorbance spectra of 4-MO (**a**), CR (**b**), and MB (**c**) and their and Ln(At/A0) vs. time plot (**d**) for the reduction reactions with NaBH_4_/Sn-Gd_2_O_3_@GH. The amount of the Sn-Gd_2_O_3_@GH catalyst was kept fixed (0.2 g).

In this approach, the reduction of different dyes, such as CR, 4-MO, and MB was also examined using NaBH_4_ in the presence of Sn-Gd_2_O_3_@GH catalyst. All dyes of the same concentration of (0.07 mM), were investigated following the same process as above. Figure 7 showed that CR and MO dyes were reduced within 4 and 16 min by NaBH_4_ (in presence of Sn-Gd_2_O_3_@GH) in one min interval at room temperature, except MB which was investigated for 18 min showing no progress in reduction. The corresponding rate of reduction of the CR and MO was reported as 0.842 and 0.197 min^−1^, respectively. The reduction rate was linearly associated with Equation (1). Therefore, the catalytic reduction with NaBH_4_ (In presence of Sn-Gd_2_O_3_@GH catalyst) was reported efficient for CR dye.

#### 4.1.3. Catalytic Reduction Changing Experimental Parameter

The industrial development causes a serious threat to human beings owing to the release of huge number of pollutants, especially dyes, to direct stream flow. These dyes’ molecules contain double bond azo group (–N=N–) in their skeleton of chemical structure [66,67]. Synthetic dyes are used for various purposes, mainly in the cosmetic, food, pulp, paper, and textile industries. In particular, CR and MO are both anionic azo dyes, which are used as indicators as well as in the food, textile, paper and pulp, cotton, silk, plastic, cosmetic, pharmaceutical, rubber, printing, and wool industries [68]. Despite the useful applications of CR, it also causes eye and skin irritation, gastro-intestinal irritation, and vomiting, while its long-term subjection to the body causes liver tumours in women [69]. CR dye is also used as a biological stain in medicine for diagnosis of amyloidosis and an indicator in acidic media [70,71].

**Figure 8:**
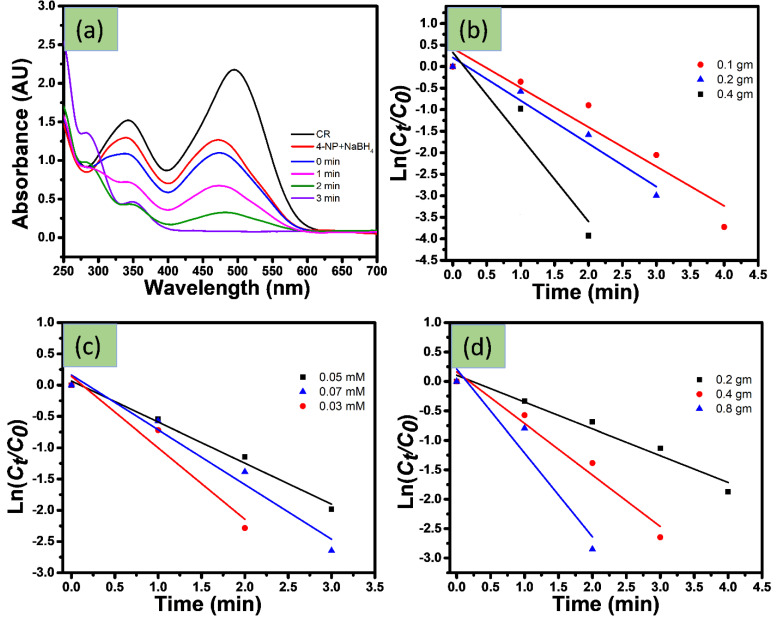
UV–visible spectra of CR dye reduction with NaBH_4_/Sn-Gd_2_O_3_@GH (**a**). Optimization of CR dye degradation. The plot of ln (Ct/C0) versus time for CR dye by changing amount of Sn-Gd_2_O_3_@GH catalyst (**b**). Different concentrations of CR dye (**c**) and changing the amount of NaBH4 (**d**).

Therefore, the catalytic reduction of CR dye was carried out with strong reducing agent (NaBH_4_) and Sn-Gd_2_O_3_@GH, with Sn-Gd_2_O_3_@GH nanocomposite as the catalyst. For the spectral reduction of CR dye in the presence of NaBH_4_/Sn-Gd_2_O_3_@GH, the UV–visible spectrometer was found and presented in Figure 8a.

The UV–Vis spectrum of reduction of CR was recorded for 4 min in the presence of NaBH_4_, using 0.2 gm of Sn-Gd_2_O_3_@GH catalyst (Figure 8a). With the introduction of NaBH_4_ to the aqueous medium of CR, the absorption peak at 495 nm was reduced to absorption band (λ _max_) at 475 nm.

In the reaction mixture of CR+NaBH_4_, the concentration of NaBH_4_ was kept higher, which resulted in the increase in the pH of the reaction mixture. The addition of hydrogel nanocomposite (Sn-Gd_2_O_3_@GH catalyst) helps in catalytic reduction by transferring electrons from electron donor species BH_4_‾ to electron acceptor species CR. The catalyst decreases the activation energy and stabilize the reaction mixture [72]. As the reaction mixture is independent on the concentration of the NaBH_4_, and hence, for the catalytic reduction of the present mixture a pseudo first order kinetics could be used. Therefore, the linear relationship between ln(*C_t_/C*_0_) (where *A*_0_ is absorbance of the initial concentration, *A_t_* is the absorbance at the specific reaction time) vs. time confirmed that the reaction is of the pseudo-first order. Further, the efficiency of Sn-Gd_2_O_3_@GH was further optimized and analysed against different catalyst amounts (Sn-Gd_2_O_3_@GH), NaBH_4_ amounts, and CR concentrations.

The change in reduction rate was further analysed, changing 0.1, 0.2, and 0.4 gm of Sn-Gd_2_O_3_@GH catalyst against CR, in the presence of 0.4 gm NaBH_4_. The UV–vis absorbance was recorded in 1 min intervals. For each run, 25.0 mL (0.07 mM) CR was introduced into the beaker, followed by the addition of 0.4 gm NaBH_4_, adding 0.1, 0.2, or 0.4 gm catalyst, respectively. That widely records the decrease in reduction time of the dye by increasing catalyst amount. The reduction of the CR was complete in 4, 3, and 2 min with the reduction rate of 0.915, 0.999, and 1.96 min^−1^, respectively. This clearly demonstrates that the reduction time decreases with the increase in the catalyst amount, as 0.4g of Sn-Gd_2_O_3_@GH reduced the CR in 2 min with reduction rate of 1.96 min^−1^, as shown in Figure 8b. To determine the catalyst efficiency against different concentrations of the dye, three different concentrations, 0.07, 0.05, and 0.03 mM of the CR dye were prepared. Each concentration of 25.0 mL was consecutively introduced into a 50.0 mL beaker using 0.4 gm of NaBH_4_ and 0.2g of the prepared catalyst. The CR was reduced in 3, 3, and 2 min with a corresponding rate of 0.875, 0.656, and 1.14 min^−1^, respectively. The 0.03 mM was reduced with high reduction rate of 1.14 min^−1^ as shown in Figure 8c. Similarly, Figure 8d represents the effect of NaBH_4_ for the reduction of 0.07 mM CR. Herein, three different weights of 0.2, 0.4, and 0.8 gm of NaBH_4_ were added to 25 mL of the (0.07 mM) dye using 0.2 gm of the catalyst. By the transfer of the above NaBH_4_ amount to the beaker in the presence of catalyst, the dye was reduced in 4, 3, and 3 min with reduction rate of 0.455, 0.875, and 1.42 min^−1^, respectively. Again, the higher reduction rate of 1.42 min^−1^ confirmed the influence of NaBH_4_ on the catalytic efficiency of Sn-Gd_2_O_3_@GH nanocomposite. The apparent rate constant (K_app_) of the pseudo order kinetic for all changing parameters is shown in (Figure 8b–d) between ln*(C_t_/C_0_)* and time, which were fitted with the above linear Equation (1). Similarly, the % reduction in the dyes was calculated using the given Equation (2).

Reduction (%) *C/C*_0_ = [(*A*_0_ − *A_t_*)/*A*_0_] * 100 (2)

where *A*_0_ and *A_t_* are corresponding to the initial absorbance of the dye and absorbance at time *‘t’*, respectively.

## 5. Mechanism of Reduction

**Figure 9:**
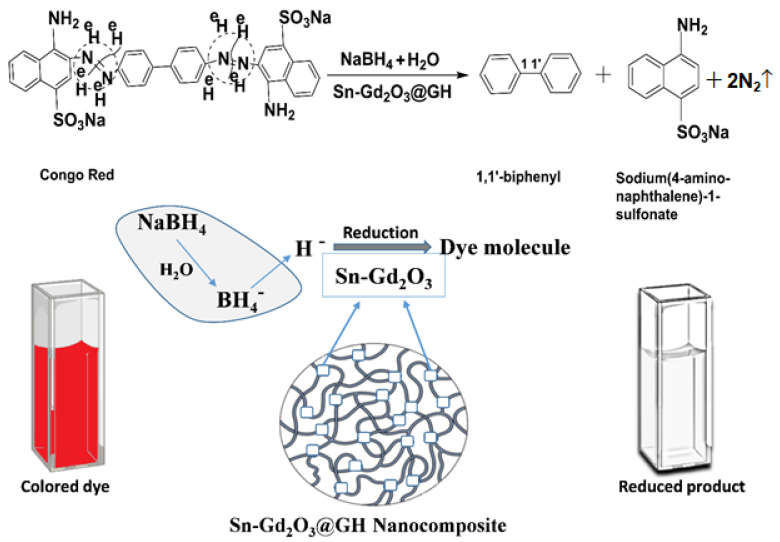
Scheme for the reduction of the CR by NaBH4 using Sn-Gd_2_O_3_@GH hydrogel catalyst.

CR is a di-azo dye widely used in different industries. Besides its importance, it is also hazardous to nature. Therefore, from an environmental point of view, its reduction is absolutely paramount. Its chemical structure is composed of two phenyl groups which are connected by two diazo binding to the two naphthalene on the both side of the biphenyl group [73]. Reduction of CR molecules cannot be carried out by NaBH_4_ because this reaction is not feasible kinetically. Therefore, here, the reduction of CR is carried out by Sn-Gd_2_O_3_@GH nanocomposite with NaBH_4_. Figure 9 shows the reduction of CR, which is converted into its corresponding constituents. Before introducing the catalyst to the reaction media, a high concentration of NaBH_4_ was employed to the aqueous solution of CR, which raised the pH of the solution and changed its colour from dark red to lighter red. The NaBH_4_ gives BH_4_^-^ and acts as an electron donor. By introducing the Sn-Gd_2_O_3_@GH catalyst, the BH_4_^-^ and dye molecules adsorb on the surface of the catalyst. The surface of the nanomaterial acts as mediator for transferring electrons from borohydride ions (BH_4_^-^) to CR molecules. The catalyst provides an efficient pathway to transfer electrons to CR molecules and reduced it in a small amount of time. Finally, the CR molecule was broken down into 1,1’-bipheny, sodium(4-amino-naphthalene)-1-sulfonate, and nitrogen.

## 6. Reusability of Catalyst

**Figure 10:**
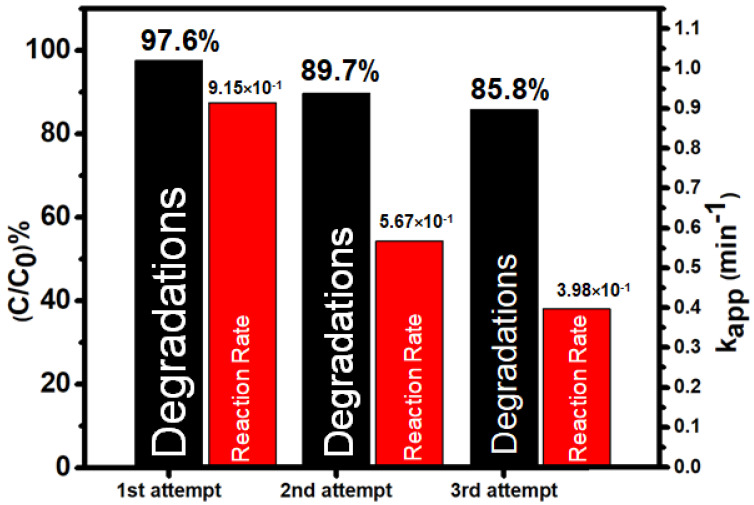
Recyclability of the Sn-Gd_2_O_3_@GH hydrogel catalyst. Degradation (red) and reaction rate (black) histograms are shown for the reusability of Sn-Gd_2_O_3_@GH catalyst during CR degradation in the presence of borohydrate.

Here, the important factor of the efficient catalyst is its recyclability property [74]. The recyclability determines the durability and performance of the catalyst. Here, the Sn-Gd_2_O_3_@GH catalyst was checked for three cycles after successful completion of the reaction. Figure 10 exhibits the C/C_0_ (%) and k_app_ of three catalytic reduction reactions of CR dye. The catalyst was easily recovered from the reaction mixture simply by filtration and then washed with distilled water and reused again.

It is clearly observed form Figure 10 that CR was reduced by Sn-Gd_2_O_3_@GH with sodium borohydrate in 4, 5, and 5 min, respectively, up to 97.6, 89.7, and 85.8 %, with catalytic rate of 9.15 × 10^−1^, 5.67 × 10^−1^, and 3.98 × 10^−1^ min^−1^, respectively. The concentration of NaBH_4_ was kept constant throughout the recyclability. For each cycle, the recovered amount of Sn-Gd_2_O_3_@GH hydrogel catalyst was used against 0.7 mM solution of CR. A slight decrease was exhibited in the activity of the catalyst with reusability.

## 7. Conclusions

Sn doped Gd_2_O_3_ nanomaterial was successfully prepared. The nanomaterial was simply prepared by mixing of 0.1 M solution of Gd[NO_3_]_3_·6H_2_O and SnCl_4_ at PH > 10. The solution was heated in a Teflon-lined autoclave at 150 °C to obtain the white precipitate of the sample. Then, 0.4 % by weight of the white precipitate of Sn-Gd_2_O_3_ was treated with 20 mL of gelatin solution and cross-linked with formaldehyde, and finally Sn-Gd_2_O_3_@GH nanocomposite was synthesized. The hydrogel nanocomposite was further used as a catalyst against different dyes, such as 4-NP, 2,6-DNP, 2-NP, MB, MO, and CR, in the presence of strong reducing agent NaBH_4_. The Sn-Gd_2_O_3_@GH catalyst reduced the CR efficiently with respect to other dyes, with a 9.15 × 10^−1^ min^−1^ rate of reaction. The XRD pattern confirms the tetragonal rutile phase of the crystal. The SEM micrographs evaluate the average crystalline size of Sn-Gd_2_O_3_nanomaterial, which is calculated as 30.06 nm, while EDS peaks confirm the incorporated material in the hydrogel nanocomposite. Therefore, the present approach leads to the fabrication of a new hydrogel nanocomposite for the removal of water pollutants, which is efficient for the reduction activity of dyes and can be easily recycled and reused.

## Data Availability

Data will be available upon reasonable request.
